# PVT1 Exon 9: A Potential Biomarker of Aggressive Prostate Cancer?

**DOI:** 10.3390/ijerph13010012

**Published:** 2015-12-22

**Authors:** Adeodat Ilboudo, Jyoti Chouhan, Brian K. McNeil, Joseph R. Osborne, Olorunseun O. Ogunwobi

**Affiliations:** 1 Department of Biological Sciences, Hunter College, The City University of New York, New York, NY 10065, USA; ailboudo@genectr.hunter.cuny.edu (A.I.); 2 Department of Urology, State University of New York Downstate Medical Center, New York, NY 11203, USA; jyoti.d.chouhan@gmail.com (J.C.); Brian.McNeil@downstate.edu (B.K.M.); 3 Department of Radiology, Memorial Sloan Kettering Cancer Center, New York, NY 10065, USA; osbornej@mskcc.org; 4 Joan and Sanford I. Weill Department of Medicine, Weill Cornell Medical College, Cornell University, New York, NY 10065, USA

**Keywords:** PVT1 exon 9, prostate cancer, disparity, biomarker, males of African ancestry

## Abstract

Prostate cancer (PCa) is the most commonly diagnosed cancer as well as the greatest source of cancer-related mortality in males of African ancestry (MoAA). Interestingly, this has been shown to be associated with single nucleotide polymorphisms around regions 2 and 3 of the 8q24 human chromosomal region. The non-protein coding gene locus Plasmacytoma Variant Translocation 1 (PVT1) is located at 8q24 and is overexpressed in PCa and, therefore, is also a candidate biomarker to explain the well-known disparity in this group. PVT1 has at least 12 exons that make separate transcripts which may have different functions, all of which are at present unknown in PCa. Our aim was to determine if any PVT1 transcripts play a role in aggressiveness and racial disparity in PCa. We used a panel of seven PCa cell lines including three derived from MoAA. Ribonucleic acid extraction, complementary deoxyribonucleic acid synthesis, and quantitative polymerase chain reaction (qPCR) were performed to evaluate expression of all 12 PVT1 exons. Each qPCR was performed in quadruplicates. At least four separate qPCR experiments were performed. Expression of PVT1 exons was inconsistent except for exon 9. There was no significant difference in exon 9 expression between cell lines derived from Caucasian males (CM), and an indolent cell line derived from MoAA. However, exon 9 expression in the aggressive MDA PCa 2b and E006AA-hT cell lines derived from MoAA was significantly higher than in other cell lines. Consequently, we observed differential expression of exon 9 of PVT1 in a manner that suggests that PVT1 exon 9 may be associated with aggressive PCa in MoAA.

## 1. Introduction

Prostate cancer (PCa) is the most common non-cutaneous cancer and the second leading cause of cancer-related death for men in the U.S. It is estimated that, in 2015, approximately 220,800 new cases of PCa will be diagnosed and 27,540 deaths will result from PCa [1]. African Americans have the highest incidence of PCa in the world, with an annual average of 229 per 100,000 men for the period of 2006–2010, which represents about two-fold more than Caucasian Americans [2]. PCa is also the leading cancer in terms of incidence and mortality in men from Africa and the Caribbean [3]. Consequently, African ancestry is a very important risk factor.

PCa is a heterogeneous disease, with multiple risks factors. The specific reasons for poor outcomes from PCa in males of African ancestry (MoAA) when compared to Caucasian males (CM) are not understood. However, it is widely believed that the causes of PCa disparities are complex and multifaceted. Two potential reasons are frequently proposed to explain this profound disparity in PCa: (1) MoAA present more often than CM with advanced incurable PCa due to more limited access to health care [4,5,6]; (2) PCa is biologically more aggressive in MoAA than CM, and can be attributed to environmental and/or genetic risk factors.

The 8q24 human chromosomal region is one of the most important susceptibility genetic loci for PCa. Several studies have identified single nucleotide polymorphisms (SNPs) located in chromosome 8q24 as susceptibility markers for PCa [7,8,9,10,11,12]. The 8q24 chromosomal region has only one protein-coding gene, the well-known MYC oncogene implicated in different cancers, including PCa. However, it also has a number of non-protein coding genes (such as PVT1) whose functional roles have not been thoroughly investigated yet [13].

In recent years, non-protein coding RNAs (ncRNAs) have received special attention because they have been identified in many studies as being important in cancer biology. Substantial progress has been made in understanding the role of small non-coding RNAs such as microRNAs (miRNAs) in the development and progression of cancers [14,15]. However, studying the role of long non-coding RNAs (lncRNAs) in cancers appears to be more complicated. LncRNAs are defined as endogenous cellular RNAs that have a size of more than 200 nucleotides, and that do not possess an extended open reading frame [16,17,18,19].

**Figure 1:**
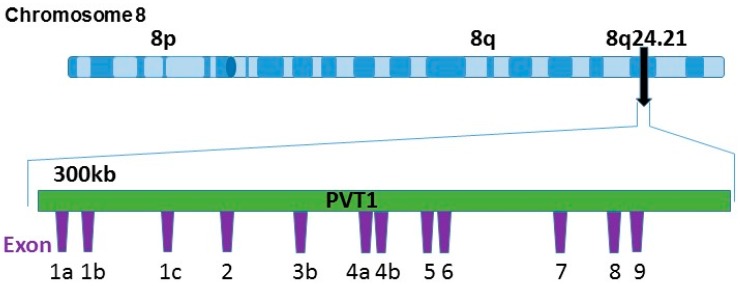
PVT1 localization on chromosome 8q24. PVT1 is a 300 kb long non-protein coding gene locus found at the 8q24 human chromosomal region. The PVT1 gene locus comprises at least 12 annotated exons.

Plasmacytoma variant translocation 1 (PVT1) is one of the transcribed lncRNAs located at the 8q24 PCa susceptibility locus (Figure 1). The PVT1 gene locus expresses several alternatively spliced non-protein coding transcripts [20,21,22] and it also encodes a cluster of microRNAs [23]. However, no specific functional role for any of these transcripts has been identified [9,24]. PVT1 is located downstream of MYC. It has a size of over 300 kb and, since its discovery in the mid-80s [25,26], it has been proven to play an important role in cancer. The upregulation of PVT1 has been found to be involved in poor prognosis in colorectal cancer and gastric cancer [27,28]. In non-small cell lung cancer, it promotes tumorigenesis [29]. In PCa, PVT1 has been found to have an increased expression in comparison to normal prostate tissue with the presence of a newly identified functional PCa specific genetic variant, rs378854 [30]. PVT1 encodes for several transcripts, approximately 12 exons, and their differential expression has not previously been investigated. Consequently, in the present study, we report data on the relative expression of the 12 different exons of PVT1 in models representative of a wide variety of clinical PCa. Our observations indicate that exon 9 of PVT1 is significantly overexpressed in PCa derived from aggressive PCa in MoAA, thus suggesting the potential for clinical utility in this population group least served by current management strategies.

## 2. Materials and Methods

### 2.1. Cell Lines

For this study, seven prostate epithelial cell lines were used, in representation of the heterogeneity notable in clinical PCa. The RWPE1 cells are epithelial cells derived from the peripheral zone of a histologically normal prostate from a 54-year-old CM. The cells were later transfected with a single copy of the human papilloma virus 18 (HPV-18) to finally establish the cell line. RWPE1 is non-tumorigenic. WPE1-NA22 cells were obtained by exposing RWPE1 to *N*-methyl-*N*-nitrosourea. WPE1-NA22 cells are mildly tumorigenic. MDA PCa 2b cells were derived from bone metastasis of prostate adenocarcinoma in a 64-year-old MoAA. PC-3 was derived from bone metastasis of a grade IV prostatic adenocarcinoma from a 62-year-old CM. The VCaP cell line was established from a vertebral bone metastasis from a 59-year-old CM with hormone refractory PCa. The E006AA cell line was established in 2004 by Koochekpour *et al.* [31], from a 50-year-old MoAA who underwent radical retropubic prostatectomy for treatment of clinically-localized PCa. The E006AA cell line is non-tumorigenic in nude mice. The highly tumorigenic derivative of E006AA, the E006AA-hT cell line, was established and characterized in 2014. The main features of all the cell lines used in this study are summarized in Table 1.

All the cell lines were purchased from the American Type Culture Collection (ATCC, Manassas, VA, USA) except for the E006AA and E006AA-hT cell lines that were a kind gift from Dr. Shariar Koochekpour of the Roswell Park Cancer Institute (New York, USA).

**Table 1:** Main characteristics of prostate epithelial cell lines used in this study.

Cell line	Caucasian Male (CM)	Male of African Ancestry (MoAA)	Indolent Disease	Aggressive Disease	Normal Prostate	Androgen Status
RWPE1	√				√	Dependent
WPE1-NA22	√		√			Dependent
MDA PCa 2b		√		√		Dependent
E006AA		√	√			Independent
E006AA-hT		√		√		Independent
PC-3	√			√		Independent
VCaP	√			√		Dependent

### 2.2. Cell Culture and Cell Culture Reagents

RWPE1 and WPE1-NA22 cells were maintained in Keratinocyte-Serum Free Medium (Life Technologies, Grand Island, NY, USA) supplemented with 0.05 mg/mL bovine pituitary extract and 5 ng/mL human Epidermal Growth Factor. MDA PCa 2b cells were maintained in F-12K medium supplemented with 20% fetal bovine serum (FBS), 25 ng/mL cholera toxin, 10 ng/mL mouse epidermal growth factor, 0.005 mM phosphoethanolamine, 100 pg/mL hydrocortisone, 45 nM selenous acid and 0.005 mg/mL bovine insulin. PC-3 cells were maintained in F-12K medium supplemented with 10% FBS. VCaP cells were maintained in DMEM medium supplemented with 10% FBS. E006AA and E006AA-hT cells were maintained in DMEM medium supplemented with 10% FBS. All cell lines were also cultured with the presence of 1% Penicillin/Streptomycin.

### 2.3. Primer Design and Sequences

Prior to our study, the following PVT1 exons were described: 1a, 2, 3b, 4b, 7, 8 and 9 [13]. However, using the UCSC Genome browser, we carefully annotated the PVT1 sequence and 12 exons were retrieved from the analysis. The Primers3 Plus software [32] was used to custom-design primers for all 12 annotated exons, and the sequences of the primers are listed in Table 2.

**Table 2:** List of primer sequences of PVT1 exons; * patent pending.

Primer Name	Primer Sequence 5′-3′
PVT1 Exon 1A-F	ACGAGCTGCGAGCAAAGA
PVT1 Exon 1A-R	CGTGTCTCCACAGGTCACAG
PVT1 Exon 1B-F	CGGAAGCTGCAGAAGGACAAA
PVT1 Exon 1B-R	CTCAAATAATGGAGACCAGGCCA
PVT1 Exon 1C-F	GCAGTGCAGGAAGCCAACTA
PVT1 Exon 1C-F	CTTAGGGGTCCTTACAGCCAAG
PVT1 Exon 2-F	AACCATGCACTGGAATGACA
PVT1 Exon 2-R	CATCAGATGCTTCACCAGGA
PVT1 Exon 3B-F	CATACTCCCTGGAGCCTTCTC
PVT1 Exon 3B-R	CAGTGTCCTGGCAGTAAAAGG
PVT1 Exon 4A-F	GGGTTCAAGTGATCCTCCTG
PVT1 Exon 4A-R	TGTAATCCCAGCACGTTGAA
PVT1 Exon 4B-F	CACCTGGGATTTAGGCACTT
PVT1 Exon 4B-R	CCAATCTCAAAATACTCCAGCTTT
PVT1 Exon 5-F	GCCAACAGAGATTTTGAGAAACAC
PVT1 Exon 5-R	TCAGCTCAGGTTCCCATTGT
PVT1 Exon 6-F	TGCTAGGGTGACAGAAACTGG
PVT1 Exon 6-R	CCCAGGTCTTGATGACAGGT
PVT1 Exon 7-F	TTGGTGCTCTGTGTTCACCT
PVT1 Exon 7-R	TGTCCACTAGCAGCAACAGG
PVT1 Exon 8-F	AGAATAACGGGCTCCCAGAT
PVT1 Exon 8-R	AAGCTGGGTCTTCATCCTGA
PVT1 Exon 9-F *	CATGACTCCACCTGGACCTT
PVT1 Exon 9-R *	GTGGGCGATGAAGTTCGTA

### 2.4. RNA Extraction and RT-QPCR

At 75% confluency, total RNA was extracted from cells in a 60 × 15 mm tissue culture dish, using RNeasy Mini Kit (Qiagen, Germany, cat# 74104). After quantification with Nanodrop1000 spectrophotometer (NanoDrop, Madison, WI, USA), 1 μg of RNA was reverse-transcribed into cDNA using QuantiTect Reverse Transcription kit (Qiagen, Germany, cat# 205311). Amplification reactions were performed in 25 μL reaction volume using SYBR Green PCR master Mix (Life Technologies, Grand Island, NY, USA cat# 4309155), cDNA template and 0.4 μM final concentration for primers. The thermal cycle profile employed was as follows: 50 °C for 2 min, 10 min initial denaturation at 95 °C, and 40 cycles of 15 s denaturation at 94 °C, 1 min annealing at 65 °C. A dissociation curve was also added at the end of the cycle. The amplifications were carried out on the 7500 Real Time PCR machine (Applied Biosystems instruments, Grand Island, NY, USA). Messenger RNA (mRNA) expression was assessed in quadruplicates in at least 3 independent experiments and normalized to RPL32 mRNA expression. Relative expression levels were calculated by the Ct method (∆∆ Ct). Previously published RPL32 primer sequences were used [31].

### 2.5. Statistical Analysis

Data are presented as mean ± standard error of the mean (S.E.M) of at least three independent experiments. Statistical significance of differences was assessed using two-tailed Student’s *t* test. *p* values less than 0.05 were deemed significant. A summary of all *p* values resulting from comparing each prostate cell line to the normal prostate cell line RWPE1 for all PVT1 exons are summarized in Table 3.

**Table 3:** Summary of p values comparing each prostate cell line to the normal prostate cell line RWPE1 for all PVT1 exons.

PVT1 exon	WPE1-NA22	MDA PCa 2b	E006AA	E006AA-hT	PC-3	VCaP
**PVT1 Exon 1A**	0.4148	0.1017			0.2865	0.0262
**PVT1 Exon 1B**	0.2950	0.0133			0.0060	0.3158
**PVT1 Exon 1C**	0.2864	0.2326			0.0686	0.0369
**PVT1 Exon 2**	0.3284	0.0016			0.0114	0.2598
**PVT1 Exon 3B**	0.4073	0.4516			0.0359	0.1179
**PVT1 Exon 4A**	0.4410	0.0010	0.3320	0.2397	0.1518	0.0633
**PVT1 Exon 4B**	0.0780	0.4943			0.0130	0.4064
**PVT1 Exon 5**	0.1867	0.0244			0.3641	0.0938
**PVT1 Exon 6**	0.1160	0.4997			0.3313	0.0409
**PVT1 Exon 7**	0.1976	0.1799			0.3066	0.1383
**PVT1 Exon 8**	0.1492	0.2963			0.0255	0.0650
**PVT1 Exon 9**	0.0179	0.0050	0.0742	0.0416	0.0007	0.3552

## 3. Results

We sought to assess the expression of 12 annotated PVT1 exons in various prostate cancer cell lines. Initial experiments focused on identifying exons that may be significantly overexpressed or underexpressed in PCa cell lines derived from MoAA.

**Figure 2:**
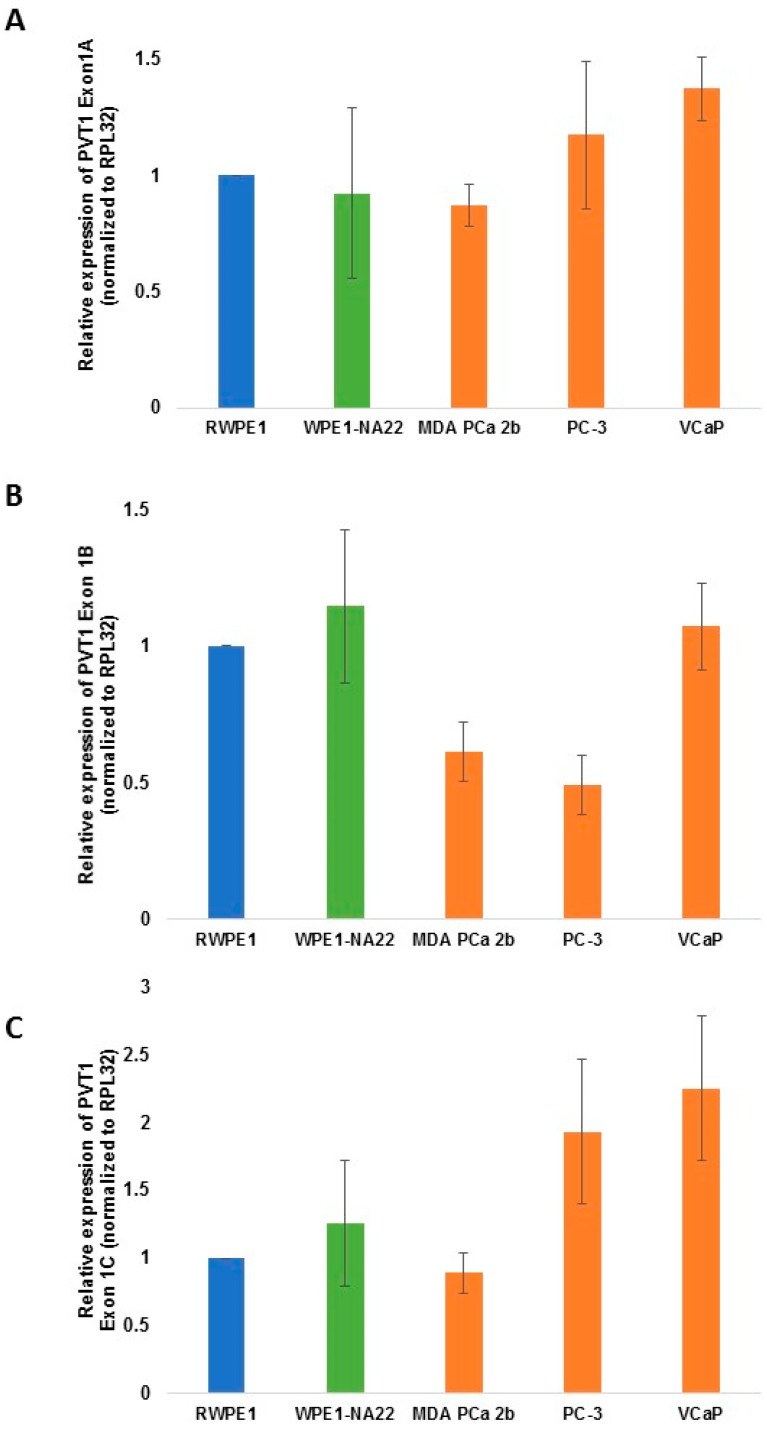
PVT1 exons 1a (**A**); 1b (**B**) and 1c (**C**) expression in non-tumorigenic and tumorigenic prostate epithelial cell lines. Four independent qPCR experiments were performed and every experiment was set up in quadruplicates. The data showed that there is no significant differential expression of the exons in the cell lines in comparing cell lines derived from CM with those derived from MoAA. The data are presented as mean + standard error of the mean (SEM).

For PVT1 exon 1a and 1c, no significant differential expression was observed in the MDA PCa 2b PCA cell line derived from a male of African ancestry, when compared to the RWPE1 normal prostate epithelial cell line (Figure 2). For PVT1 exon 1b, a decrease in the expression level was observed in the MDA PCa 2b cell line, but it was not significant (Figure 2).

**Figure 3:**
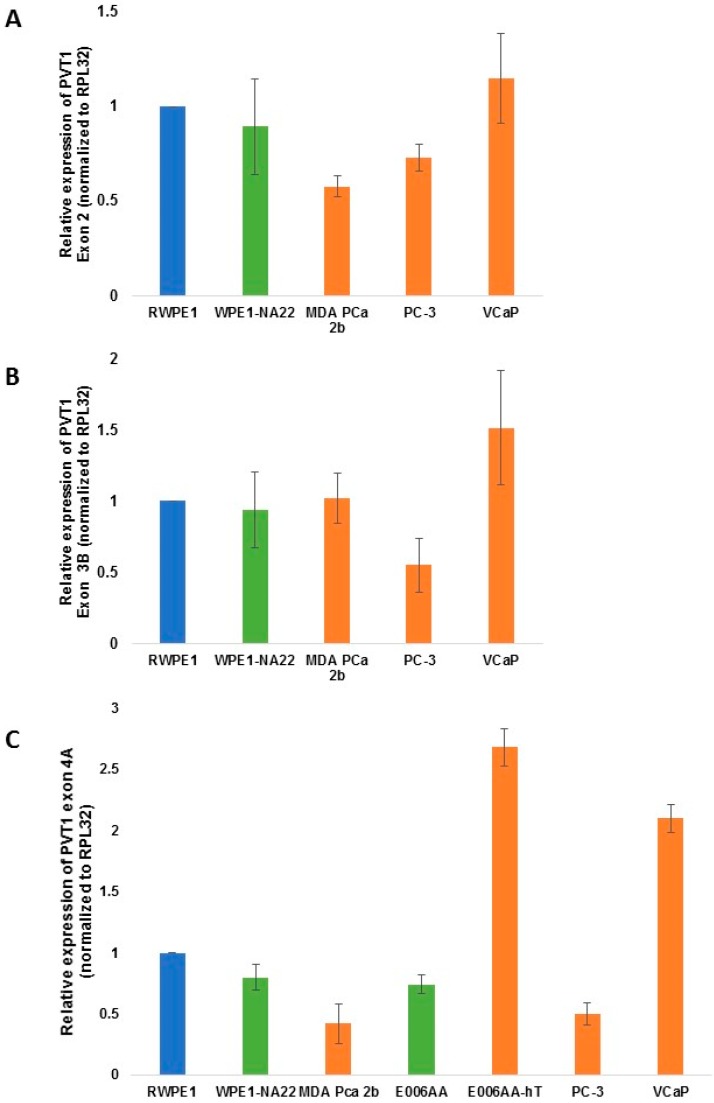
PVT1 exons 2 (**A**); 3b (**B**); and 4a (**C**) expression in non-tumorigenic and tumorigenic prostate epithelial cell lines. Four (for exon 2 and exon 3b) or 3 (for exon 4a) independent qPCR experiments were performed and every experiment was set up in quadruplicates. The data showed that there is no significant differential expression of the exons in the cell lines in comparing cell lines derived from CM with those derived from MoAA. The data are presented as mean + standard error of the mean (SEM).

For PVT1 exon 2, we observed a small but significant decrease (*p* value = 0.00161) in relative expression by MDA PCa 2b in comparison to RWPE1 (Figure 3). However, for PVT1 exon 3b, no difference in relative expression was observed when MDA PCa 2b was compared to RWPE1 (Figure 3). For PVT1 exon 4a, a significant decrease in relative expression by MDA PCa 2b of almost 60% in comparison to RWPE1 was observed (*p* value = 0.001104). However, PVT1 Exon 4a was overexpressed in the highly tumorigenic E006AA-hT also derived from a MoAA (Figure 3). However, this overexpression was not statistically significant (*p* value = 0.2397). Given that both MDA PCa 2b and E006AA-hT are highly tumorigenic and derived from MoAA, the dissimilar expression of PVT1 exon 4a in them suggests inconsistency and possible lack of importance.

**Figure 4:**
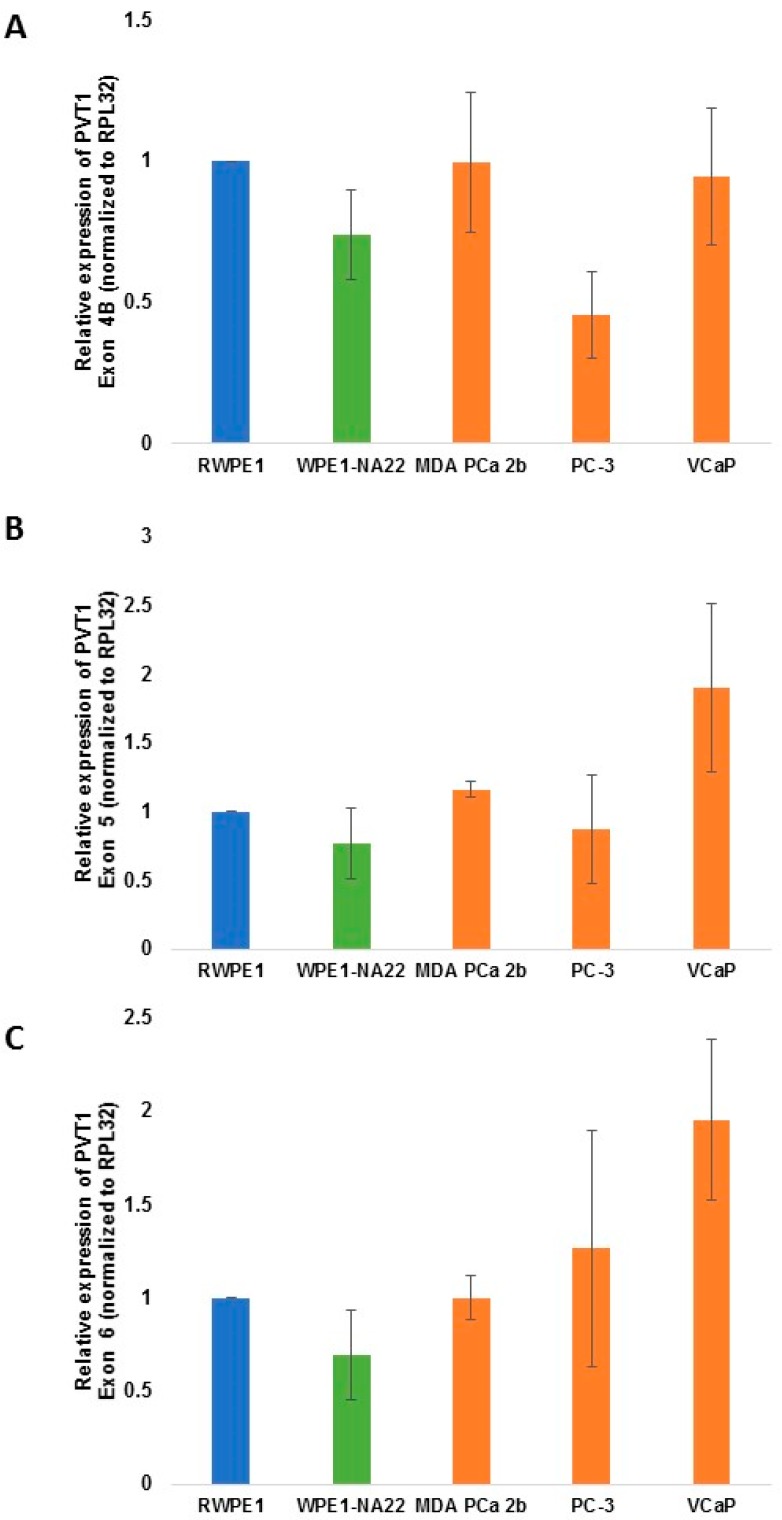
PVT1 exons 4b (**A**); 5 (**B**) and 6 (**C**) expression in non-tumorigenic and tumorigenic prostate epithelial cell lines. Four independent qPCR experiments were performed and every experiment was set up in quadruplicates. The data showed that there is no significant differential expression of the exons in the cell lines in comparing cell lines derived from CM with those derived from MoAA. The data are presented as mean + standard error of the mean (SEM).

**Figure 5:**
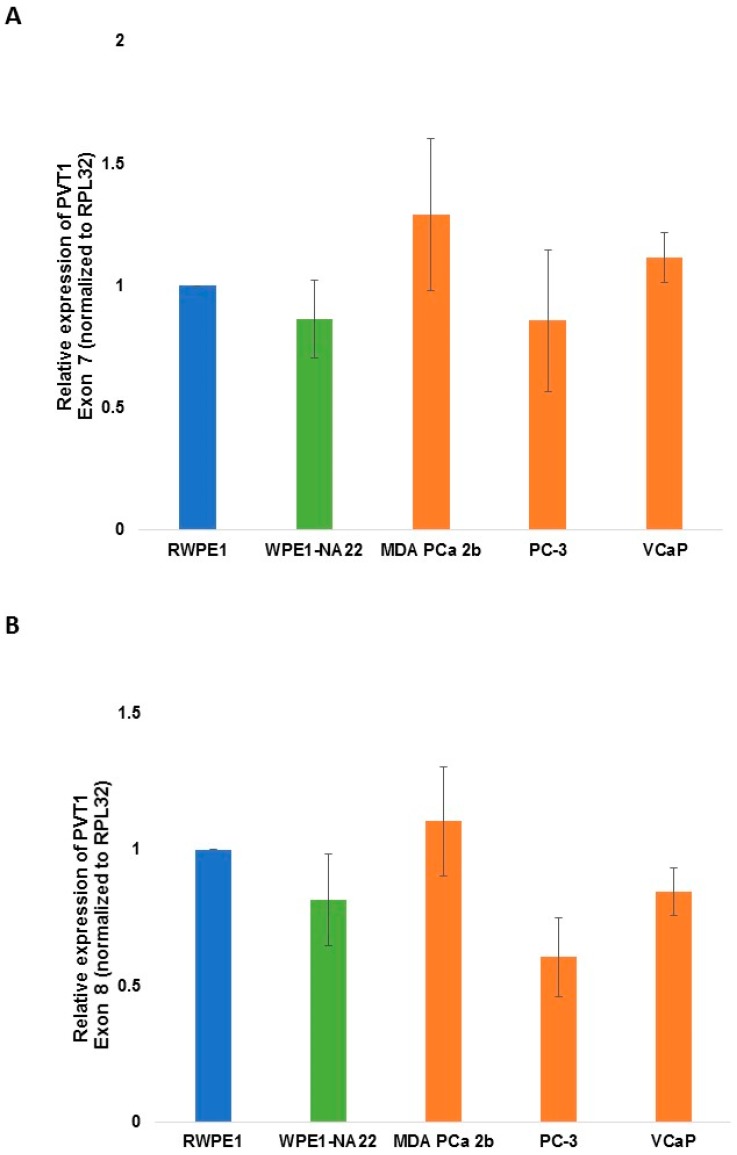
PVT1 exons 7 (**A**) and 8 (**B**) expression in non-tumorigenic and tumorigenic prostate epithelial cell lines. Four independent qPCR experiments were performed and every experiment was set up in quadruplicates. The data showed that there is no significant differential expression of the exons in the cell lines in comparing cell lines derived from CM with those derived from MoAA. The data are presented as mean + standard error of the mean (SEM).

For PVT1 exons 4b, 5, 6 (Figure 4), 7 and 8 (Figure 5), there were no differential expression observed in comparing MDA PCa 2b to RWPE1.

**Figure 6:**
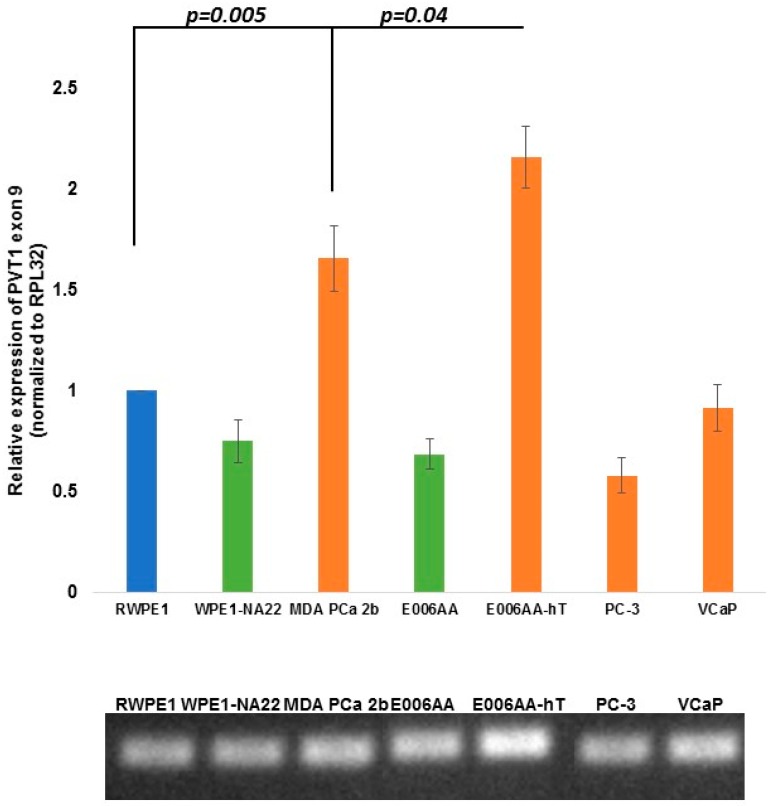
PVT1 exon 9 overexpression in aggressively tumorigenic PCa cell line from MoAA. Five independent qPCR experiments were performed and every experiment was set up in quadruplicates. The data showed that PVT1 exon 9 is consistently significantly overexpressed in the aggressive PCa cell lines derived from MoAA in comparison to the non-tumorigenic PCa cell line it was derived from. The data are presented as mean + standard error of the mean (SEM) and the *p* values are displayed on the figure. The lower panel shows the different prostate cell lines’ PVT1 exon 9 PCR products loaded on a 0.8% agarose gel.

**Figure 7:**
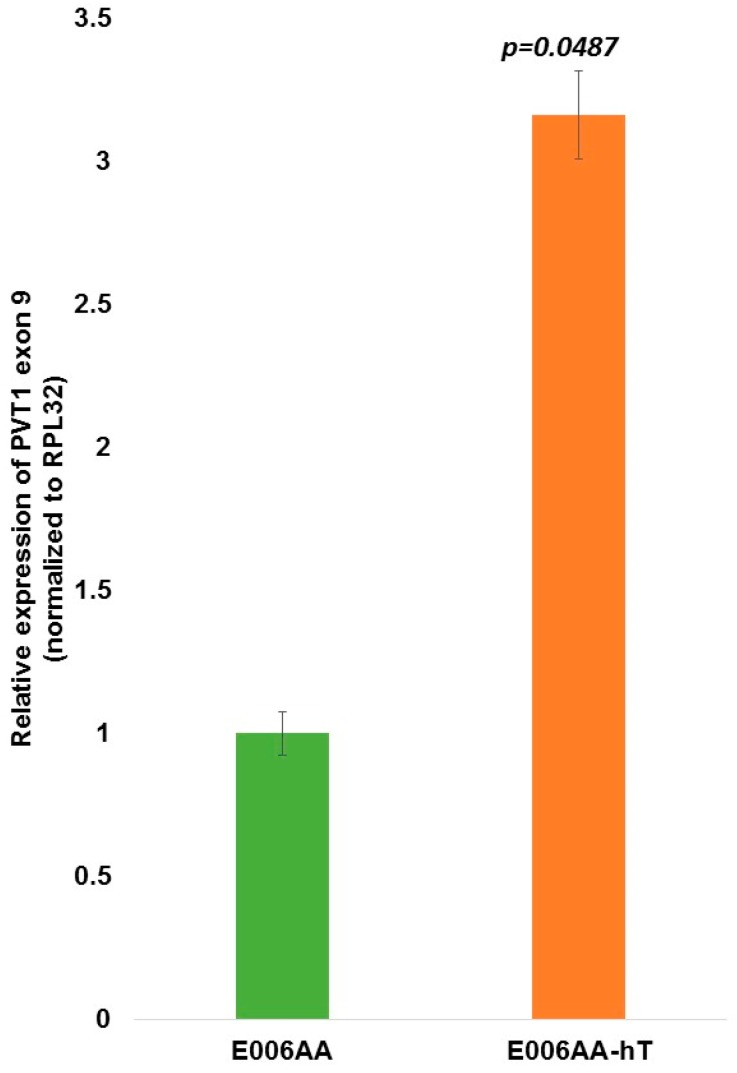
PVT1 exon 9 expression in non-tumorigenic and tumorigenic prostate epithelial cell lines. At least three independent qPCR experiments were performed and every experiment was set up in quadruplicates. The data showed that PVT1 exon 9 is consistently significantly overexpressed in the aggressive PCA cell lines derived from MoAA. The data are presented as mean + standard error of the mean (SEM) and the p values are displayed on the figure, and are compared to the control (RWPE1 cell line).

PVT1 exon 9 was the only one of all 12 exons of PVT1 that showed a very consistent and easily explainable expression profile. PVT1 exon 9 was significantly and consistently overexpressed in both aggressively tumorigenic cell lines derived from men of African ancestry. In both MDA PCa 2b and E006AA-hT cell lines, relative expression of PVT1 exon 9 in comparison to the RWPE1 cell line were approximately 200% (two-fold) higher (Figure 6). Interestingly, in comparing the E006AA non-tumorigenic cell line derived from a MoAA with its derivative, the aggressively tumorigenic E006AA-hT cell line, PVT1 exon 9 expression was about 300% higher in the E006AA-hT cell line (*p* = 0.0487; Figure 7). This indicates that PVT1 exon 9 is related to aggressiveness in this model of PCa in a MoAA.

## 4. Discussion

The long non-protein coding RNA (lncRNA) PVT1 has been shown to be important in cancer. PVT1 overexpression has been demonstrated in pancreatic cancer and colon cancer, and is related to poor prognosis in most of these cases [27,28,29]. However, these studies did not distinguish between the different transcripts of PVT1. It is possible that the different exons of PVT1 could be differentially expressed, and have different functions. Here, we report for the first time a study where we annotated and designed primers for amplification of 12 separate exons of PVT1. Our results show that most of the exons do not have differential expression in PCa. However, very interestingly, one particular exon of PVT1, PVT1 exon 9, was consistently and significantly overexpressed in the aggressive PCa cell lines derived from MoAA. Importantly, we demonstrate that PVT1 exon 9 is significantly overexpressed in the aggressive PCa cell line derived from a MoAA in comparison to its non-tumorigenic cell line from which it was derived. This clearly indicates that PVT1 exon 9 overexpression is significantly associated with aggressiveness in this model of PCa in a MoAA. Whether this will translate to individuals or subpopulations at risk prospectively is at present unknown. A more in depth evaluation of prospectively collected PCa gene databases and fresh prostate specimens will be required to understand whether this phenomenon can be used as a biomarker for MoAA with PCa.

To determine if a lncRNA is important, its cellular functions need to be elucidated. It will be important to determine if the lncRNA regulates important cellular functions or if it just represents “transcriptional noise” or background transcription. Although lncRNAs are sometimes aberrantly expressed in diseased tissues, suggesting specific functions in diseases, our knowledge of how these lncRNA act in the cell and which roles they might play in diseases in still very limited [32,33]. Therefore, an understanding of the cellular functions, mechanisms of action, and mechanisms regulating PVT1 exon 9 expression will be critical to exploiting it for potential clinical applications.

Our results show that PVT1 exon 9 is consistently overexpressed in aggressively tumorigenic PCa cell lines derived from MoAA. This observed overexpression of PVT1 exon 9 in the aggressive PCa cell lines derived from MoAA suggests that PVT1 exon 9 may contribute to the high proportion of PCa aggressiveness in MoAA. To better understand the cellular and molecular mechanisms of this contribution, we need to understand the functional role of PVT1 exon 9 in PCa development and progression. To this end, its expression pattern in human PCa with varied clinico-pathological characteristics, its cellular functions, its molecular mechanisms of action, and its molecular targets will need to be determined. Some of this work has started to be done in other cancers. For example, in 2014, Takahashi *et al.* reported that the amplification of PVT1 was involved in poor prognosis via the inhibition of apoptosis in colon cancers [27].

Assessing for a functional role for PVT1 exon 9 could be done by studying the effects of silencing of PVT1 transcripts using the RNAi approach. In this regard, Gray and colleagues were able to induce apoptosis in ovarian or breast cancer cell lines by silencing of PVT1 in cell lines with amplified chr.8q24 but they found no apoptosis in non-amplified cell lines [34]. Similar studies assessing cellular function will need to be performed in PCa. Understanding the role of PVT1 exon 9 in PCa aggressiveness in MoAA may lead to the future possibility of exploiting PVT1 exon 9 for diagnosis, therapy, and other clinical applications in PCa.

## 5. Conclusions

The present study reports the overexpression of PVT1 exon 9 by aggressively tumorigenic PCa cell lines derived from MoAA. Based on this consistent overexpression of PVT1 exon 9 by aggressive PCa cell lines derived from MoAA, it is possible that PVT1 exon 9 is important and may contribute to the disproportionately increased aggressiveness of PCa in MoAA.
